# Unidirectional recruitment between MeCP2 and KSHV-encoded LANA revealed by CRISPR/Cas9 recruitment assay

**DOI:** 10.1371/journal.ppat.1012972

**Published:** 2025-03-10

**Authors:** Ido Lavi, Supriya Bhattacharya, Ankita Awase, Ola Orgil, Nir Avital, Guy Journo, Vyacheslav Gurevich, Meir Shamay

**Affiliations:** Daniella Lee Casper Laboratory in Viral Oncology, Azrieli Faculty of Medicine, Bar-Ilan University, Safed, Israel; The University of North Carolina at Chapel Hill School of Medicine, UNITED STATES OF AMERICA

## Abstract

Kaposi’s sarcoma-associated herpesvirus (KSHV, HHV-8) is associated with several human malignancies. During latency, the viral genomes reside in the nucleus of infected cells as large non-integrated plasmids, known as episomes. To ensure episome maintenance, the latency protein LANA tethers the viral episomes to the cell chromosomes during cell division. Directional recruitment of protein complexes is critical for the proper function of many nuclear processes. To test for recruitment directionality between LANA and cellular proteins, we directed LANA via catalytically inactive Cas9 (dCas9) to a repeat sequence to obtain easily detectable dots. Then, the recruitment of nuclear proteins to these dots can be evaluated. We termed this assay CRISPR-PITA for Protein Interaction and Telomere Recruitment Assay. Using this protein recruitment assay, we found that LANA recruits its known interactors ORC2 and SIN3A. Interestingly, LANA was unable to recruit MeCP2, but MeCP2 recruited LANA. Both LANA and histone deacetylase 1 (HDAC1) interact with the transcriptional-repression domain (TRD) and the methyl-CpG-binding domain (MBD) of MeCP2. Similar to LANA, HDAC1 was unable to recruit MeCP2. While heterochromatin protein 1 (HP1), which interacts with the N-terminal of MeCP2, can recruit MeCP2. We propose that available interacting domains force this recruitment directionality. We hypothesized that the tandem repeats in the SunTag may force MeCP2 dimerization and mimic the form of DNA-bound MeCP2. Indeed, providing only the tandem epitopes of SunTag allows LANA to recruit MeCP2 in infected cells. Therefore, CRISPR-PITA revealed the rules of unidirectional recruitment and allowed us to break this directionality.

## Author summary

Using a CRISPR/Cas9 recruitment assay, we show that some interacting proteins have a unidirectional recruitment property, where only one of the proteins can recruit its partner. We found unidirectional recruitment relations between the methylated DNA binding protein MeCP2 and Kaposi’s sarcoma-associated herpesvirus (KSHV)-encoded LANA. Where MeCP2 recruits LANA, but LANA is unable to recruit MeCP2. We hypothesized that the tandem repeats in the SunTag may force MeCP2 dimerization and mimic the form of DNA-bound MeCP2. Indeed, providing only the tandem epitopes of SunTag allows LANA to recruit MeCP2 in infected cells. We propose that this unidirectional recruitment is the result of available interacting domains.

## Introduction

Kaposi’s sarcoma-associated herpesvirus (KSHV, HHV-8) is the causative agent of all forms of Kaposi’s sarcoma (KS) and is tightly associated with primary effusion lymphoma (PEL) and multicentric Castleman’s disease [1,2]. Like all herpes viruses, KSHV has two phases: latent (dormant) and lytic (productive) cycle. During latency, the viral genomes reside in the nucleus of infected cells as large non-integrated plasmids, known as viral episomes. All KSHV-infected cells express LANA, and LANA is essential for viral latency [3–5]. LANA binding to the viral episomes is critical both for replication by recruitment of the cellular replication machinery to the viral genomes [6,7], and for maintenance by tethering the viral episomes to the cell chromosomes during cell division [8–10]. Both the N and C-terminal regions of LANA have been shown to mediate chromosome association; the N-terminal via binding to core histones H2A and H2B [11], while for the C-terminal, several candidates were identified, including MeCP2, DEK, and RING3 [12–14]. Multiple LANA binding sites (LBS1, LBS2, and LBS3) [15,16] within the KSHV terminal repeats, and its oligomerization ability [17,18] create visible LANA dots in KSHV-infected cells. In contrast, LANA is equally distributed in the nucleus when expressed in un-infected cells [10,12,13,19]. So, it is challenging to determine whether LANA or the viral genomes are the recruiters of cellular factors.

For many nuclear processes, directional recruitment of protein complexes is critical for proper function. Transcription factor that binds a specific DNA sequence recruit co-activators and general transcription factors that result in subsequent recruitment of RNA polymerase leading to gene expression. In this scenario, a transcription factor is expected to recruit the co-activator and not vice versa. One example of recruitment directionality is the estrogen receptor, where the combination of ligand binding, dimerization, and sequence-specific DNA binding are required to generate a high-affinity surface to recruit co-activator proteins [20]. This conformational change upon DNA binding and dimerization will favor the binding and recruitment of co-activators to enhancer/promoter-bound receptors and minimize interaction with DNA-free receptors.

MeCP2 binds methylated DNA and recruits co-repressor complexes to execute the readout of CpG methylation into transcription repression [21]. Several studies detected an interaction between MeCP2 and histone deacetylase 1 (HDAC1). Recruitment of histone deacetylase activity plays a role in transcription repression imposed by MeCP2 [22,23]. MeCP2 and HDAC1 represent an example of two proteins that interact in the nucleus. While MeCP2 recruits HDAC1 to methylated regions to repress transcription, we expect that HDAC1 should not recruit MeCP2 to promoters in those cases where HDAC1 is recruited by other transcription factors. Another protein that plays an important role in transcription repression is heterochromatin protein 1 (HP1). HP1 interacts with the repressive mark histone three lysine 9 tri-methylation (H3K9Me3), and via recruitment of SUV39H1, the histone H3K9Me3 methyltransferase, leads to the spreading of this heterochromatin repressive histone mark [24,25]. HP1 has been shown to interact with MeCP2 [26,27], but whether HP1 can recruit MeCP2 is an open question.

Cas9 is an endonuclease that can be directed to different DNA targets that are complimentary to a guide RNA and contain a PAM sequence [28]. Soon after the discovery of this property, many applications of this sequence-specific targeting of Cas9 emerged. The endonuclease is no longer needed for targeting purposes, and a catalytically dead mutant Cas9 (dCas9) that still retains DNA binding capability was created [28,29]. Targeting this dCas9 to promoter sequences results in transcription activation or repression depending on the fused domains and the targeting location relative to the transcription start site [29,30]. Shortly after the creation of the dCas9, it was directed to repeat elements to visualize these elements in fixed [31] and live cells [32–34].

Here, we harness CRISPR/Cas9 to determine recruitment relations between LANA and cellular proteins. We termed this method CRISPR-PITA for Protein Interaction and Telomere recruitment Assay. We combined the ability of the SunTag system [35] to gather up to ten molecules of a protein of interest with CRISPR/dCas9 targeted to a repeat sequence such as telomeres. The ability of this protein to recruit endogenous or fluorescently tagged proteins to these telomere dots is determined. Interestingly, we found that recruitment does not always work bi-directionally. LANA fails to recruit MeCP2, but MeCP2 efficiently recruits LANA. Similarly, HDAC1, which interacts with the transcriptional-repression domain (TRD) of MeCP2, same as LANA, was unable to recruit MeCP2. While HP1α, which interacts with the N-terminal of MeCP2, was able to recruit MeCP2. We hypothesized that the interaction domain with LANA will become available for interaction only upon DNA binding and dimerization, and proposed that the tandem repeats in the SunTag may force MeCP2 dimerization and mimic the form of DNA-bound MeCP2. Indeed, providing only the tandem epitopes of SunTag allows LANA to recruit MeCP2 in infected cells. Therefore, CRISPR-PITA revealed the rules of unidirectional recruitment and allowed us to break this directionality.

## Results

### Creation of visible dots at the telomeres with dCas9-SunTag and scFv-LANA

**Fig 1:**
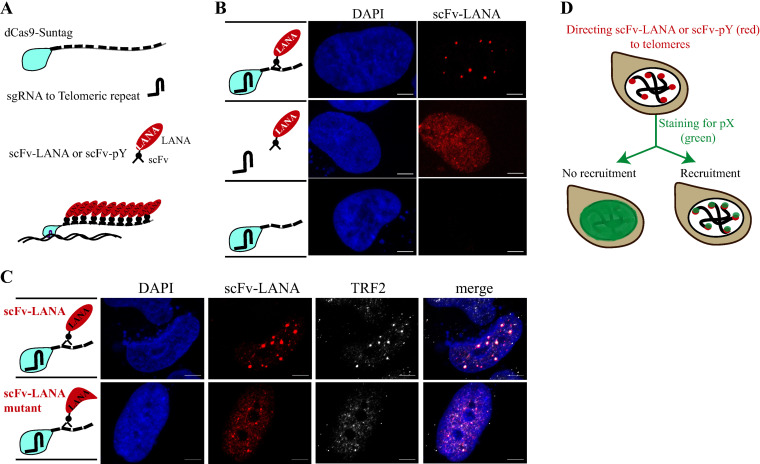
Schematic illustration of CRISPR-PITA. **(A)** Schematic illustration of dCas9-SunTag, sgRNA, and scFv-LANA or other protein of interest (scFv-pY). **(B)** SLK cells were transfected with dCas9-SunTag, scFv-LANA, and sgTelomere, as illustrated on the left of the images. An immunofluorescence assay detected LANA (red), and the nucleus was stained with DAPI (blue). Scale bar = 5 μm. Images are representatives of at least three independent experiments. **(C)** SLK cells were transfected with dCas9-SunTag, sgTelomere, and scFv-LANA or scFv-LANA oligomerization mutant, as illustrated on the left of the images. An immunofluorescence assay detected LANA (red) and TRF2 (white). The nucleus was stained with DAPI. Images are representatives of at least two independent experiments. **(D)** Schematic illustration of the CRISPR-PITA, targeting of scFv-LANA or any other protein of interest to the telomeres via dCas9. Then, the recruitment of other nuclear proteins to these dots is evaluated via immunostaining.

The KSHV-encoded LANA localizes in large dots together with the viral episomes in KSHV-infected cells but is equally distributed in the nucleus when expressed in un-infected cells [10,12,13,19], and Fig 1B (middle panel). To test recruitment directionality between LANA and nuclear proteins we were interested to create LANA dots in un-infected cells. The ability of dCas9 to direct proteins of interest to any genomic locus makes it an ideal system for a recruitment assay. To enhance dot formation, we utilized the SunTag system, where tandem epitopes can concentrate multiple molecules of an antibody-fused protein that recognizes this epitope (up to 10 with the tag used in this study) [35]. In our case, the tandem epitopes are fused to dCas9, and the antibody scFv is fused to our protein of interest (Fig 1A). Transfection of LANA fused with scFv (scFv-LANA) together with dCas9 and sgRNA for telomere repeat sequence, resulted in LANA-telomere-dots that were visible by immuno-fluorescence assay (IFA) for LANA (Fig 1B). The correct size of scFv-LANA and other scFv-fused proteins used in this study were validated by Western blotting (S1 Fig). When the dCas9 was omitted from the mix, LANA could not generate nuclear dots, suggesting that LANA-telomere-dots depend on the targeting of LANA to telomeres via dCas9. To validate that LANA was targeted to the telomeres, we used an antibody against the telomeric repeat factor 2 (TRF2), and indeed scFv-LANA co-localized with TRF2 when co-transfected with dCas9 and sgTelomere (Fig 1C). The ability of LANA to oligomerize [15,17,18,36,37] may explain the large dots by facilitating the recruitment of additional dCas9-SunTag to the same telomere via LANA-LANA interaction or by bringing together several telomeres. Studies of the LANA oligomerization property identified a LANA oligomerization mutant [17,18]. Therefore, we created this LANA oligomerization mutant (F1037A/F1041A) in the context of scFv-LANA (scFv-LANA-mutant) and compared the dot size to scFv-LANA. As expected, the LANA oligomerization mutant created a larger number of small telomere dots compared to wt LANA for both LANA and TRF2 (Fig 1C). These experiments indicate that we can generate LANA dots in un-infected cells using the dCas9-SunTag system.

### LANA recruits ORC2 and SIN3A but not MeCP2

**Fig 2:**
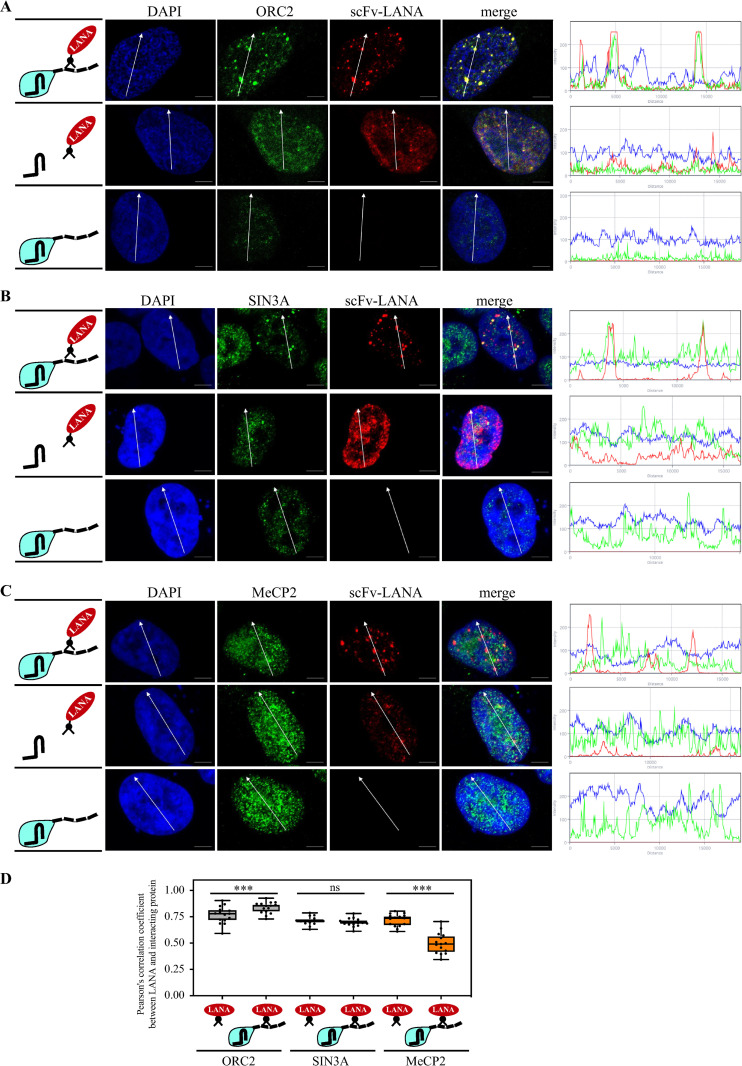
LANA recruits ORC2 and SIN3A but not MeCP2. SLK cells were transfected with dCas9-SunTag, scFv-LANA, and sgTelomere expression plasmids, as indicated in the illustrations on the left. Immunofluorescence assays detected LANA (red) and ORC2 **(A)** mSin3a **(B)** or MeCP2 **(C)** (in green) cellular localization. The nucleus was stained with DAPI. Scale bar = 5 μm. Images are representatives of at least three independent experiments. The plots of the red, green, and blue pixel intensities along the white arrow (in the middle panels) are presented. **(D)** Pearson’s correlation coefficient was determined by ImageJ (JACoP Pluing) [62] for 15 cells in each treatment and presented as box and whiskers (min to max). Two-tailed *t* tests were performed (*, **P** ≤ 0.05; **, **P** ≤ 0.01; ***; **P** ≤ 0.001).

In this assay, a protein of interest is targeted to telomeres via dCas9-SunTag, and the recruitment of endogenous proteins to these dots is detected by immunofluorescence assay (Fig 1D). We termed this method CRISPR-PITA for Protein Interaction and Telomere recruitment Assay. We performed the CRISPR-PITA assay for ORC2, a subunit of the Origin Replication Complex (ORC), previously reported to interact with LANA and localize with LANA-episome-dots [18,38–40]. ORC2 was recruited by LANA, as was determined by the easily detected dots that co-localized with LANA (Fig 2A) and the overlapping fluorescence intensity peaks along the arrow line (on the right side of the images). It is important to note that while the dCas9-SunTag and scFv-LANA were ectopically expressed, the recruited proteins are endogenous, indicating the ability of LANA to recruit the endogenous ORC2.

Both the corepressor protein SIN3A (mSin3a) [40,41] and the methyl-CpG binding protein MeCP2 [12,13,42] have been reported to interact with LANA. However, MeCP2 is not associated with LANA dots in KSHV-infected cells [43]. We found that scFv-LANA recruited SIN3A to LANA-telomere-dots, both by the visual observation of the dots and the fluorescence intensity plot (Fig 2B). Analysis of MeCP2 recruitment via the CRISPR-PITA revealed no recruitment of MeCP2 to LANA-telomere-dots (Fig 2C). We also determined the Pearson’s correlation coefficient for the different treatments (Fig 2D). Since these proteins are known LANA interactors, we expected to find overlap when LANA alone (without dCas9) is transfected. When LANA is targeted to the telomeres via dCas9, if LANA can recruit its partner, the overlap is expected to remain or even increase. In contrast, if LANA cannot recruit its partner, the overlap should be reduced. This is exactly what we found when we measured the Pearson’s correlation coefficient for 15 cells in each experiment. The overlap was reduced for the non-recruited MeCP2, remained the same for SIN3A, and increased for ORC2.

One possibility to explain the lack of MeCP2 recruitment by LANA is that as a methylated DNA binding protein, it is engaged in binding methylated DNA and, therefore, not available to be recruited by LANA. To test this, we performed the CRISPR-PITA in cells with knock-out (KO) of DNMT1 and DNMT3b (HCT DKO) that were reported to lose over 95% of their CpG methylation [44]. Even in HCT DKO, scFv-LANA could not recruit MeCP2 to LANA-telomere-dots (S2 Fig), suggesting that protein availability could not explain the lack of recruitment in this case. Immunofluorescence assays for ORC2, SIN3A, and MeCP2 in KSHV-infected PEL (BCBL1) cells revealed that while ORC2 and SIN3A were associated with LANA-episome-dots, MeCP2 was not associated with LANA-episome-dots (S3 Fig), supporting the ability of the CRISPR-PITA to predict recruitment ability between proteins.

### MeCP2 recruits LANA

**Fig 3:**
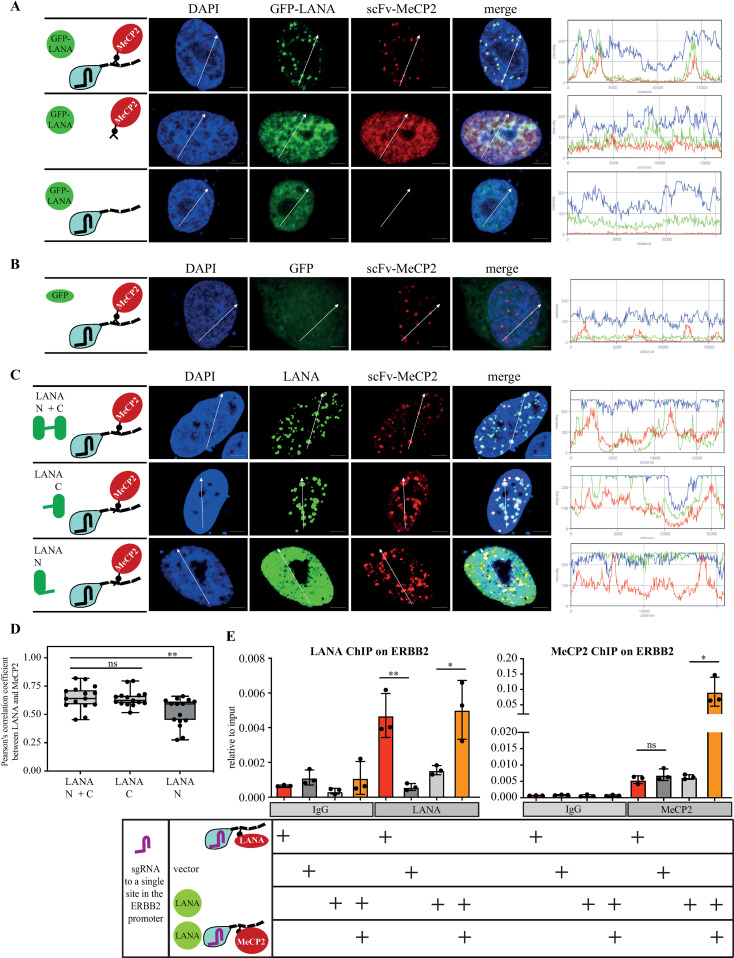
MeCP2 recruits LANA. SLK cells were transfected with dCas9-SunTag, scFv-MeCP2, and sgTelomere in combination with GFP-LANA **(A)** or GFP **(B)**, as illustrated on the left. An immunofluorescence assay detected scFv-MeCP2, or fluorescently labeled GFP-LANA and GFP. The nucleus was stained with DAPI. Scale bar = 5 μm. Images are representatives of at least three independent experiments. The plots of the red, green, and blue pixel intensities along the white arrow (in the middle panels) are presented. **(C)** SLK cells were transfected with FLAG-LANA N+C, FLAG-LANA C, or FLAG-LANA N, the same as in A. An immunofluorescence assay was performed to detect both scFv-MeCP2 and LANA. **(D)** Pearson’s correlation coefficient was determined for 15 cells in each treatment and presented as box and whiskers (min to max). Two-tailed *t* tests were performed (*, **P** ≤ 0.05; **, **P** ≤ 0.01; ***; **P** ≤ 0.001). **(E)** ChIP assay was performed on HEK293 cells to detect LANA (left panel) and MeCP2 (right panel) association with the ERBB2 promoter following recruitment with sgRNA to this locus.

The observation that scFv-LANA could not recruit MeCP2 despite their documented interaction [12,13,42] highlights the importance of determining recruitment relations between proteins. Krithivas et al. have shown that the expression of human MeCP2 can recruit LANA to heterochromatin in NIH 3T3 mouse cells [13]. Therefore, we tested the ability of scFv-MeCP2 to recruit LANA to MeCP2-telomere-dots. Cells were co-transfected with dCas9, sgTelomere, scFv-MeCP2, and expression vectors for GFP-LANA or GFP alone as a control. The scFv-MeCP2 was able to recruit GFP-LANA to MeCP2-telomere-dots, but not GFP alone, supporting the ability of MeCP2 to recruit LANA (Fig 3A, B). LANA contains three distinct domains: the N-terminal region (AA 1-329), the C-terminal (AA 936-1162), and a middle repeat region. Both the N and C-terminal regions have been shown to mediate chromosome association; the N-terminal via binding to core histones H2A and H2B [11], while for the C-terminal, several candidates were identified, including MeCP2, DEK, and RING3 [12–14]. To determine the LANA protein domain that is recruited by MeCP2, we performed CRISPR-PITA by scFv-MeCP2 for LANA N+C, LANA N, and LANA C. While MeCP2 efficiently recruited both LANA N+C and LANA C, no recruitment was observed with the N-terminal (Fig 3C, D). This result agrees with a previous study that located the interaction between LANA and MeCP2 in the C-terminal region [12]. Importantly, this result further supports the notion that despite the high concentration of the recruiter protein at telomeres, CRISPR-PITA still requires specific interaction, as both the N-terminal of LANA and GFP-alone were not recruited by MeCP2 in our assay.

Targeting dCas9 to a repeat element results in high protein concentration at specific nuclear locations. One concern that might be raised is whether the recruitment we observed with CRISPR-PITA is specific or an artifact of the high protein concentration. To rule out this possibility, we directed dCas9 to a single chromosomal location (at ERBB2 promoter) and followed the association of the proteins with this location by chromatin immunoprecipitation (ChIP) assay followed by qPCR (Fig 3E). Here again we found that scFv-MeCP2 recruited more LANA to this promoter, while scFv-LANA failed to do the same for MeCP2. This result indicates that despite targeting dCas9 to a repeat sequence, CRISPR-PITA recruitment nicely reflects the recruitment by a single locus. This set of experiments highlights the distinction between interaction and recruitment. While LANA and MeCP2 were shown to interact with each other, still, MeCP2 can recruit LANA, but LANA cannot recruit MeCP2.

### T158M mutant MeCP2 fails to recruit LANA to heterochromatin

**Fig 4:**
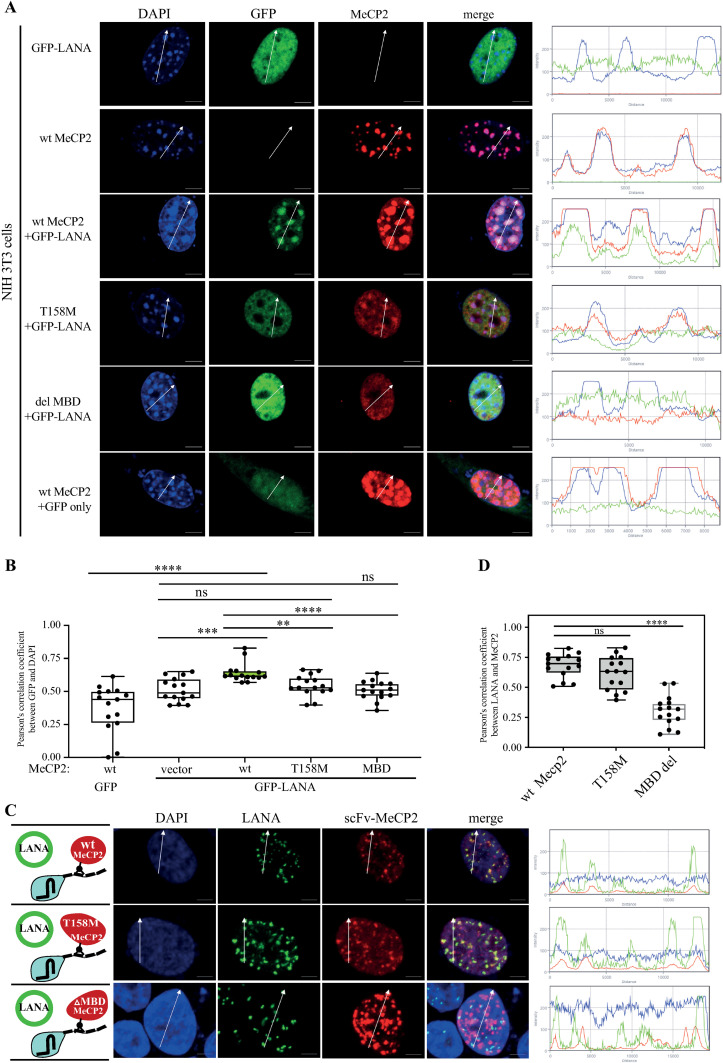
MeCP2 T158M mutant fails to recruit LANA to heterochromatin in NIH3T3 cells but can recruit LANA when artificially tethered to DNA. **(A,** **B)** NIH 3T3 cells were transfected with GFP-LANA and wt MeCP2, T158M mutant, or del-MBD. An immunofluorescence assay detected LANA (green) and MeCP2 (red). The heterochromatin foci in the nucleus of NIH3T3 mouse cells have a high intensity of DAPI (blue). Scale bar = 5 μm. Images are representatives of at least two independent experiments. The plots of the red, green, and blue pixel intensities along the white arrow (in the middle panels) are presented. **(B)** Pearson’s correlation coefficient was determined for 15 cells in each treatment and presented as box and whiskers (min to max). **(C, D)** rKSHV-infected iSLK cells (iSLK.Bac16) were transfected with dCas9-SunTag, scFv-MeCP2, and sgTelomere. An immunofluorescence assay was performed with rabbit anti-HA and rat anti-LANA and anti-rabbit Alexa Flour 555 and anti-rat Alexa Flour 647 antibodies, to detect scFv-MeCP2 and LANA dots in infected cells. In this case, the signal from anti-Rat Alexa Flour 647 antibody was pseudo-color to green to keep it consistent with the other CRISPR-PITA experiments. **(D)** Pearson’s correlation coefficient was determined same as in B for the experiment in C. Two-tailed *t-tests* were performed (*, **P** ≤ 0.05; **, **P** ≤ 0.01; ***; **P** ≤ 0.001, ****; **P** ≤ 0.0001).

To assay for the ability of MeCP2 to recruit LANA and to bind methylated DNA, we harnessed a specific feature of MeCP2 to localize to heterochromatin loci in mouse cells. This assay is typically performed in NIH 3T3 cells since they express very low levels of endogenous MeCP2 [13,45]. We cloned two mutants, one with deletion of the MBD and one with a known substitution T158M that disrupts DNA binding. We validated the correct size and equal expression of the mutated MeCP2 by Western blotting (S4 Fig). Similar to previous studies [12,13], we found that LANA can be efficiently recruited to heterochromatin when co-transfected with wt MeCP2. In contrast, both T158M and MBD-del mutants could not recruit LANA to heterochromatin loci (Fig 4A, B). Then, we tested if the T158M mutant can recruit LANA when artificially tethered to DNA via dCas9 in the CRISPR-PITA assay. This experiment was done both in transfected SLK cells (S5 A & B Fig), as well as in KSHV-infected cells (Fig 4C, D). We found that the MeCP2 T158M mutant can recruit LANA when it is artificially targeted to chromatin by dCas9 and forced to dimerize via the SunTag. While the MBD deletion mutant lost the ability to recruit LANA even when artificially targeted to chromatin. This indicates that the CRISPR-PITA can also be done in latently infected cells to recruit LANA-episome dots in infected cells. We conclude from these experiments that both T158M and MBD-del mutants could not recruit LANA to heterochromatin loci.

### MeCP2 recruits HDAC1, but HDAC1 is unable to recruit MeCP2

**Fig 5:**
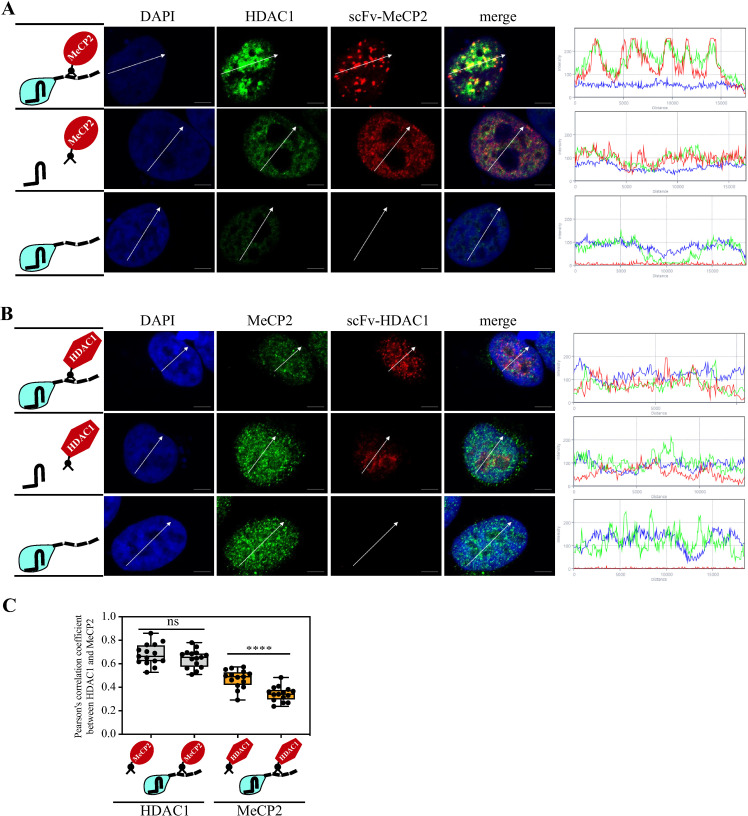
MeCP2 recruits HDAC1, while HDAC1 cannot recruit MeCP2. **(A)** As illustrated on the left, SLK cells were transfected with dCas9-SunTag, scFv-MeCP2, and sgTelomere. An immunofluorescence assay detected HDAC1 (green) and scFv-MeCP2 (red). **(B)** SLK cells were transfected the same as in A but with scFv-HDAC1. An immunofluorescence assay detected MeCP2 (green) and scFv-HDAC1 (red). The nucleus was stained with DAPI. Scale bar = 5 μm. Images are representatives of at least two independent experiments. The plots of the red, green, and blue pixel intensities along the white arrow (in the middle panels) are presented. **(C)** Pearson’s correlation coefficient was determined for 15 cells in each treatment and presented as box and whiskers (min to max). Two-tailed *t-tests* were performed (****; **P** ≤ 0.0001).

Several studies detected an interaction between MeCP2 and histone deacetylase 1 (HDAC1), and this recruitment of histone deacetylase activity plays a role in transcription repression imposed by MeCP2 [22,23]. We expect that MeCP2 recruits HDAC1 to methylated regions in order to repress transcription. However, since HDAC1 is recruited by other transcription factors to many promoters, therefore it is not expected to recruit MeCP2. We applied CRISPR-PITA to determine the recruitment relations between these two cellular proteins. We found that scFv-MeCP2 efficiently recruits HDAC1 (Fig 5A). In contrast, scFv-HDAC1 failed to recruit MeCP2 (Fig 5B, C). Thus, in the context of MeCP2 and HDAC1, recruitment is also unidirectional, enabling MeCP2 to recruit HDAC1 to repress transcription but preventing the mislocalization of MeCP2 by HDAC1.

### The availability of interacting domains determines recruitment directionality

**Fig 6:**
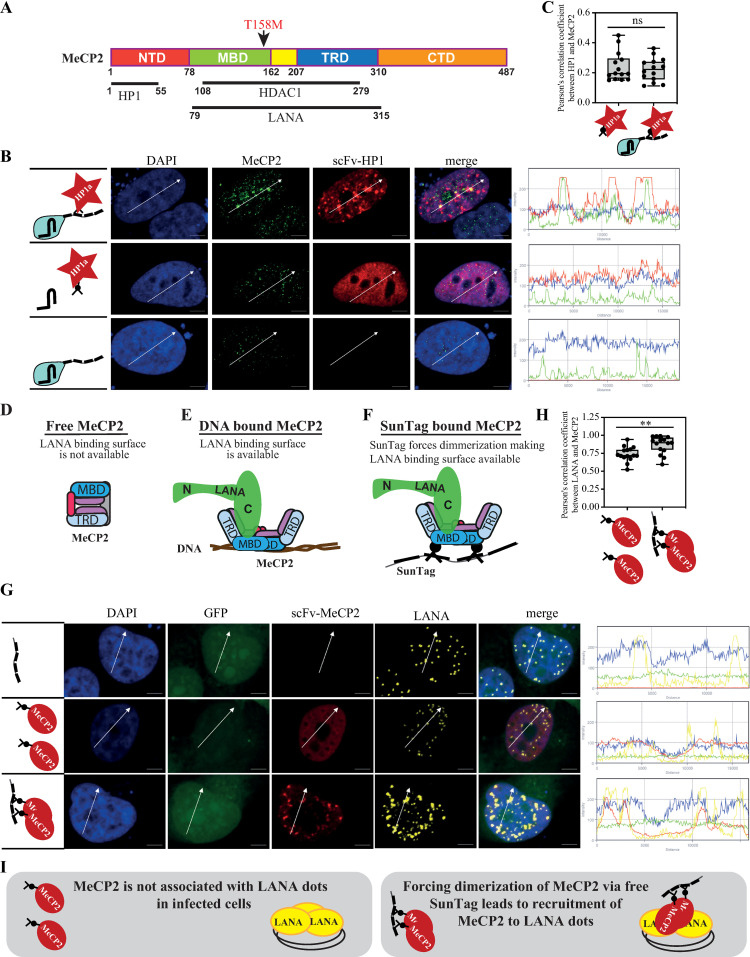
The availability of interacting domains determines recruitment directionality. (**A**) schematic presentation of MeCP2 and the interaction domains with LANA, HDAC1, and HP1α. Two Rett syndrome mutants are indicated above. **(B)** SLK cells were transfected with dCas9-SunTag, scFv-HP1α, and sgTelomere, as illustrated on the left. An immunofluorescence assay detected MeCP2 (green) and scFv-HP1α (red). The nucleus was stained with DAPI. Scale bar = 5 μm. Images are representatives of at least two independent experiments. **(C)** Pearson’s correlation coefficient was determined for 15 cells in each treatment and presented as box and whiskers (min to max). Two-tailed *t-tests* were performed. Schematic illustrations of the model for free **(D)** DNA-bound MeCP2 (**E**) and SunTag scFv-MeCP2 (**F**) are presented. (**G**) iSLK-infected cells (iSLK.Bac16) were transfected with free SunTag and scFv-MeCP2. Then, an immunofluorescence assay was performed to detect scFv-MeCP2 (red) and LANA (yellow). GFP is a marker of infected cells. The plots of the red, yellow, green, and blue pixel intensities along the white arrow (in the middle panels) are presented. **(H)** Pearson’s correlation coefficient was determined for 15 cells in each treatment and presented as box and whiskers (min to max). (**I**) Schematic presentation of the experiment in G and its conclusions.

Unidirectional recruitment between two interacting proteins is achieved when the interacting domain is available only under certain conditions. In the case of MeCP2, it is expected that the transcriptional-repression domain (TRD) will be unavailable when it is a free protein in solution and becomes available for protein interaction only when MeCP2 dimerizes upon binding to DNA (Fig 6D, E). Both LANA and HDAC1 interact with the same regions of MeCP2, the transcriptional-repression domain (TRD), and the methyl-CpG-binding domain (MBD) [36,41]. In contrast, Heterochromatin protein 1 (HP1α) interacts with the N-terminal domain (amino acids 1–55) of MeCP2 [26] (Fig 6A). We hypothesized that since HP1α interacts with a different domain that might be available for interaction also in its DNA-free state, HP1α might be able to recruit MeCP2. Indeed, we found that scFv-HP1α was able to recruit MeCP2 in CRISPR-PITA (Fig 6B, C). This result supports the notion that available interacting domains determine recruitment directionality (Fig 6D, E). A previous study [43], and our result (S3 Fig) show that LANA fails to recruit MeCP2 to LANA episome dots in infected cells. We hypothesized that forcing dimerization to DNA-free MeCP2 will break the unidirectional recruitment and enable LANA to recruit MeCP2. Although CRISPR-PITA does not directly test the DNA binding of MeCP2, however, the tandem epitopes SunTag brings several MeCP2 molecules in proximity and, therefore, might force dimerization (although we do not have direct evidence) and by that, can mimic DNA-bound MeCP2 (Fig 6F). KSHV-infected cells were transfected with scFv-MeCP2 with or without SunTag, to force MeCP2 dimerization. To exclude the possibility that the dCas9 attached to the SunTag will bind DNA even without sgRNA, we expressed the tandem epitopes SunTag only, without dCas9. Co-transfection of scFv-MeCP2 together with a SunTag resulted in the recruitment of scFv-MeCP2 to LANA dots in infected cells (**Fig 6G-I**). While the transfection of only scFv-MeCP2 could not recruit MeCP2 to LANA dots as expected. Our result that we can break the unidirectional recruitment relations between LANA and MeCP2 by forcing MeCP2 dimerization supports our proposed model of available interacting domains.

## Discussion

A powerful method to study recruitment is the Chromatin immunoprecipitation (ChIP) assay, which can detect the association of proteins with specific DNA sequences [46,47]. ChIP assays require fixation, proper shearing of the chromatin, and special antibodies that can perform the immunoprecipitation of the cross-linked chromatin. In many cases, even when two or more proteins are associated with the same genomic region, it is hard to evaluate the relationship between the proteins and determine essential factors for the recruitment. Although this limitation can be overcome by using Knockout (KO) or Knockdown (KD) of one protein and testing the ability of other proteins to be recruited to DNA/chromatin in the absence of that protein, this raises additional challenges in creating KO or KD of the target gene. Another recruitment assay is based on the integration of the *lac*O array into the genome and directing the protein of interest to this array via fusion to the Lac-repressor [48]. One limitation of this assay is the limited number of cell lines generated to contain this lacO array’s integration. In a previous study, KSHV-encoded LANA was directed to the host genome via the *lac*O array [49]. In that study, LANA recruited the two bromo and extra terminal domain (BET) proteins, BRD2 and BRD4, two known LANA interacting proteins. However, because of the system limitation, the ability of BRD2 and BRD4 to recruit LANA was not tested.

Here, we describe a simple method to test for recruitment relations between proteins. This method does not require chromatin shearing and can be performed with various antibodies that can recognize the native form of proteins. In the absence of such an antibody, the recruitment of fluorescently labeled proteins can be observed. To obtain visible dots, we combined the ability of the repeated epitope SunTag with dCas9 targeted to a repetitive genomic sequence such as the telomeres. Although targeting dCas9 to telomeres has been performed previously to follow nuclear events such as liquid–liquid phase separation (LLPS) or the generation of heterochromatin [50,51], but has not been applied to test recruitment directionality. Using this method, we found unidirectional recruitment relations between proteins. Our results with CRISPR-PITA indicated that LANA was able to recruit ORC2 and SIN3A but was unable to recruit MeCP2 to LANA-telomere-dots. While it has been shown that ORC2 localizes with LANA dots [18,38–40], and MeCP2 does not [43], there was no published data on SIN3A localization in KSHV-infected cells. So, we performed an immunofluorescence assay in KSHV-infected BCBL1 cells and found that both ORC2 and SIN3A were associated with LANA dots, but MeCP2 was not. The immunofluorescence assays in KSHV-infected cells agree with the CRISPR-PITA results, supporting the ability of CRISPR-PITA to predict the recruitment ability of proteins. While LANA recruited both ORC2 and SIN3A in CRISPR-PITA, it seems that ORC2 was recruited to most LANA dots, while SIN3A was associated only with part of LANA dots. This difference was also reflected in Pearson’s correlation coefficient, where the correlation with SIN3A remained the same while the correlation with ORC2 increased upon directing LANA to telomeres. Interestingly, this difference was also observed in infected cells, where SIN3A seems to colocalize with only part of LANA-episome dots. The recruitment of GFP-LANA by scFv-MeCP2 also suggests that CRISPR-PITA can be done without cell fixation or immunofluorescence, but directly via detection of fluorescently-labeled proteins. This property might be more relevant in cases where CRISPR-PITA is applied for screening purposes.

Targeting dCas9 to a repeat element results in high protein concentration at specific nuclear locations. One concern that might be raised is whether the recruitment we observed with CRISPR-PITA is specific or an artifact of the high protein concentration. Since directing dCas9 to a single locus (ERBB2 promoter in Fig 3E) resulted in the same recruitment directionality, rules out this possibility. Regarding specificity, we found that scFv-MeCP2 recruited the C-terminal of LANA but not its N-terminal domain. Since our recruitment directionality model is based on available interacting domains, a previous study [12] that identified the interaction domain between MeCP2 and LANA to the C-terminal domain of LANA supports our observation. The related gamma-2 herpesvirus, herpesvirus saimiri (HVS), encodes for ORF73, a functional homolog of KSHV-encoded LANA (also known as ORF73). Interestingly, also HVS encoded ORF73 interacts with MeCP2 via its C-terminal domain [45]. The lack of recruitment for the N-terminal domain of LANA indicated that recruitment is still specific and requires the interaction domain. Another support for the specific recruitment by CRISPR-PITA is the lack of recruitment observed for GFP alone. A growing list of nuclear proteins with intrinsically disordered regions and RNA/DNA binding properties can promote liquid–liquid phase separation (LLPS); both LANA and MeCP2 possess this property [52,53]. Rett syndrome MeCP2 mutants, including T158M, are impaired in LLPS formation. Since the T158M mutant can still recruit LANA in CRISPR-PITA, it excludes the possibility that recruitment in CRISPR-PITA depends on LLPS formation.

The inability of LANA to recruit MeCP2, despite their documented interaction, prompted us to test whether MeCP2 can recruit LANA. Indeed, in CRISPR-PITA, scFv-MeCP2 was able to recruit LANA. This result agrees with a previous study where MeCP2 recruited LANA to mouse heterochromatin foci [13]. This result also highlights the importance of a recruitment assay since recruitment is not always bi-directional, and, in many cases, should be functionally unidirectional. One of the LANA functions where unidirectional recruitment seems essential is LANA tethering of the viral episomal genome to the cell chromosomes during cell division. Therefore, it will be interesting in the future to test if MeCP2 has a role in the tethering of LANA and the episomes to cellular chromatin.

If LANA is recruited to MeCP2, why is MeCP2 not observed concentrated at KSHV-episomes/LANA-dots? The answer is within the directionality. While proteins that are recruited by LANA will concentrate next to the large number of LANA molecules at the episomes. On the other hand, if LANA with episomes is recruited to only a few MeCP2 molecules bound to a genomic region in a non-saturating interaction, these few MeCP2 molecules will not stand out from the rest of the MeCP2 molecules in the nucleus.

A nice example of directional recruitment is the case of MeCP2, which binds methylated DNA and recruits co-repressor complexes, including HDAC1 [22,23]. In this case, we expect that MeCP2 should recruit HDAC1, leading to transcription repression. On the other hand, HDAC1 is recruited by many transcription factors to generate repressive chromatin. Therefore, we expect that it should not recruit MeCP2 to all the chromosome locations it is recruited by other transcription repressors. Indeed, we found that scFv-MeCP2 could recruit HDAC1 to MeCP2-telomere-dots, but scFv-HDAC1 could not recruit MeCP2. Both LANA and HDAC1 interact with the MBD and TRD of MeCP2 [12,23]. In contrast, HP1α interacts with the N-terminal domain (amino acids 1–55) of MeCP2 [26]. Indeed, we found that HP1α can recruit MeCP2 to HP1α-telomere-dots. It has been shown that phosphorylation at serine 229 (pS229) of MeCP2 strongly enhances the interaction between MeCP2 and HP1α [27]. Our observation that HP1α can recruit MeCP2 may explain their observation that MeCP2 pS229 was enriched at the receptor tyrosine kinase gene *RET* promoter. In the future, it will be interesting to test the role of HP1αin the global distribution of MeCP2.

How come two proteins that interact with each other can generate unidirectional recruitment? MeCP2 is an intrinsically disordered protein that is monomeric in solution even at high concentrations but dimerizes upon DNA binding [54,55]. Although CRISPR-PITA does not directly test the DNA binding of MeCP2, however, the SunTag brings several MeCP2 molecules together and, therefore, might force dimerization. This might explain how both wt and T158M mutant MeCP2, which cannot bind DNA, can recruit LANA when dimerization is forced via the SunTag in CRISPR-PITA. To test this more directly, we forced MeCP2 dimerization with a free SunTag. Under these conditions, LANA was able to recruit MeCP2 to LANA-episome dots in infected cells. Revealing the mechanism behind unidirectional recruitment enabled us to break this unidirectionality. Our study and the CRISPR-PITA may lead to a deeper understanding of the complex formation and recruitment directionality in many cellular and viral interactions.

## Materials and methods

### Cell culture

SLK and iSLK cells (kindly provided by Don Ganem & Rolf Renne), iSLK cells infected with KSHV.Bac16 (kindly provided by Jae U. Jung), NIH 3T3 cells (kindly provided by Oren Kobiler), and HEK293 cells were cultured in Dulbecco’s modified Eagle’s medium (DMEM) supplemented with 10% fetal bovine serum (heat inactivated) and 100 U/ml penicillin, 100 μg/ml streptomycin, 2 mM L-glutamine, and 1 mM Sodium-Pyruvate in 5% CO_2_ at 37°C. HCT and HCT Dnmt1-/- Dnmt3b -/- (DKO) cells (kindly provided by Bert Vogelstein) were grown in McCoy’s 5A medium supplemented with the supplements mentioned above. BJAB and BCBL1 cells (kindly provided by Richard F. Ambinder) were cultured in RPMI-1640 medium supplemented with 20% FBS and the supplements mentioned above. Single-cell derived clonal LCLs expressing the wt or mutant Rett syndrome MeCP2 allele were established in the lab of Uta Francke [56].

### Plasmids

pHRdSV40-dCas9-10xGCN4_v4-P2A-BFP (Addgene plasmid # 60903; http://n2t.net/addgene:60903; RRID: Addgene_60903) and pHR-scFv-GCN4-sfGFP-GB1-dWPRE (Addgene plasmid # 60907; http://n2t.net/addgene:60907; RRID:Addgene_60907) were a gift from Ron Vale [35]. pgRNA-humanized was a gift from Stanley Qi (Addgene plasmid # 44248; http://n2t.net/addgene:44248; RRID: Addgene_44248) [29]. The sgRNA targeting telomeres was cloned into pgRNA-humanized. The mCherry in the pgRNA-humanized was deleted by restriction digestion and ligation. LANA, HDAC1, HP1α, and MeCP2 were PCR amplified and cloned into pHR-scFv-GCN4-sfGFP-GB1-dWPRE. scFv-LANA was further subcloned into pLIX_403, a gift from David Root (Addgene plasmid # 41395; http://n2t.net/addgene:41395; RRID: Addgene_41395). pEGFP-c2 from Clontech. GFP-LANA and LANA deletion plasmids were described previously [40,57]. For generation of MeCP2 T158M mutation, RNA was isolated from LCL 487 (T158M) cell line [56] by Qiagen RNeasy mini kit (cat no # 79656), and cDNA was prepared by maxima H minus first strand cDNA synthesis kit (cat no # K1682). A fragment of MeCP2 containing the T158M mutation was amplified by PCR and cloned in pDEST-hMeCP2-GFP (a gift from Huda Zoghbi, Addgene plasmid # 48078; http://n2t.net/addgene:48078; RRID: Addgene_48078) [58]and subsequently cloned into pHR-scFv-GCN4-sfGFP-GB1-dWPRE plasmid. MBD domain deleted MeCP2 was created by overlap extension PCR. Oligomerization mutant LANA was created by a Q5 site-directed mutagenesis kit (cat no# NEB E0554S). For ChIP assays we cloned the dCas9-5xSunTag from pCAG-dCas9-5xPlat2AflD, a gift from Izuho Hatada (Addgene plasmid # 82560; http://n2t.net/addgene:82560; RRID: Addgene_82560) [59], and sgRNA for ERBB2 promoter into lentiCRISPRv2 hygro, a gift from Brett Stringer (Addgene plasmid # 98291; http://n2t.net/addgene:98291; RRID: Addgene_98291) [60]. To generate the SunTag only (without dCas9), plasmid pHRdSV40-dCas9-10xGCN4_v4-P2A-BFP was digested by restriction enzymes MluI and BamHI, and a linker that also encode for FLAG tag was inserted. All primer sequences can be found in the supplementary primer list (S1 Table).

### Transfection and Immunofluorescence assay

1X10^5^ cells were transfected using PolyJet In Vitro DNA Transfection Reagent (SignaGene Laboratories Cat # SL100688). The pHRdSV40-dCas9-10xGCN4 v4-P2A-BFP (Addgene plasmid # 60903), scFv-proteinX, and sgRNA telomere plasmids were mixed in a 1:2:4 ratio (750 ng of total plasmid DNA). 48 h later, the cells were fixed in 4% paraformaldehyde in PBS pH 7.4 for 10 minutes, permeabilized for 10 min with PBS containing 0.25% Triton X-100. Following incubation with primary and secondary antibodies, slides were mounted with Vectaschield containing DAPI (Vector Laboratories, H-1500).

### Antibodies

The following antibodies were used for immunofluorescence studies: Rat anti-LANA (Advanced Biotechnologies, cat#13-210-100), Mouse anti-LANA (Leica, NCL-HHV8-LNA), Mouse anti-TRF2 (Merck, 05-521), Rabbit anti-MeCP2 (Abcam, ab2828), Rabbit anti-mSin3a/SIN3A (Abcam, ab3479), Mouse anti-HA (Sigma H9658), Rabbit anti-HA (ab9110), Mouse anti-ORC2 (MBL Life Science, M055-3), and Rabbit anti-HDAC1 (Abcam, ab53091). Goat anti-rabbit Alexa 488 (Abcam, ab150077), Goat anti-Rat Alexa 488 (Abcam, ab150157), Goat anti-Mouse Alexa 594 (Abcam, ab150116) and Goat anti-rabbit Alexa 555(ab27039). scFv-MeCP2, scFv-HP1α, and scFv-HDAC1 were detected with Mouse anti-HA. scFv-LANA was detected by Mouse anti-LANA, except in combination with TRF2 where Rat anti-LANA was used. scFv-MeCP2 was detected by Mouse anti-HA except for the forced dimerization experiment on SunTag in iSLK BAC16 where rabbit anti-HA was used. FLAG-LANA mutants (N+C, N, C) were detected with Rabbit anti-FLAG antibody (Sigma 7425).

### Confocal imaging

Images were captured with a Zeiss LSM780 inverted confocal microscope through a 63X objective with Z stack mode. Then, the middle stacks were selected and merged using Subset mode and Maximum Intensity Projection mode, respectively with Zenn software black edition. Processing and exporting of the resulting images was done with Zenn software blue edition.

### Chromatin immunoprecipitation assay

ChIP assays were performed as described previously [61]. Immunoprecipitation was performed with protein A/G Dynabeads (ThermoFisherScientific, #100-02D/100-04D) bound with Rat anti-LANA (Advanced Biotechnologies, cat#13-210-100), Rabbit anti-MeCP2 (Abcam, ab2828), or normal IgG (Millipore, PP64B) as a negative control. The immunoprecipitated chromatin was washed five times (with NaCl, lithium chloride, and Tris-EDTA buffers), eluted, and then purified using a QIAquick PCR purification kit (Qiagen, #28104) according to the manufacturer’s instructions.

### Western blot analysis

Cells were lysed in 0.5 ml of radioimmunoprecipitation assay (RIPA) buffer (50 mM Tris [pH 7.4], 150 mM NaCl, 1 mM EDTA, 1% Triton X-100, 0.5% sodium deoxycholate, 0.1% SDS plus protease inhibitors- Aprotinin, PMSF, leupeptine, pepstatin A (Sigma). Then the cell lysates were sonicated and cleared by centrifugation. The antibodies used for Western blot analysis included rabbit anti-HA (ab9110), Mouse anti-LANA (Leica, NCL-HHV8-LNA), mouse anti-β-actin (a2228; Sigma), Donkey anti-Rabbit HRP (Amersham, NA934V), and Sheep anti-mouse HRP (Amersham, NA931V).

### Statistics

The statistical significance of the results was calculated in Prism 9 (GraphPad). Pearson’s correlation coefficient was determined by ImageJ (JACoP Plugin) [62].

## Supporting information

S1 Fig Expression of the fused proteins. HEK 293T cells were transfected with scFv-MeCP2, scFv-MeCP2 T158M, scFv-MeCP2 delMBD, scFv-HP1, and scFv-HDAC1 (A) or scFv-LANA (B) expression vectors.
Cell extracts were subjected to SDS-PAGE and western blot analysis. The scFv-fused proteins (upper panel) and beta-actin (lower panel) were detected with anti-HA and anti-beta actin antibodies, respectively.
(PDF)

S2 Fig LANA cannot recruit MeCP2 even in HCT-DKO cells. HCT116 (A) and DKO (DNMT3B-/- and DNMT1 -/-) HCT116 cells (B) were transfected with dCas9-SunTag, scFv-LANA, and sgTelomere as illustrated on the left of the images.
An immunofluorescence assay was performed to detect MeCP2 (green) and LANA (red). The nucleus was stained with DAPI. Scale bar = 5 μm. Images are representatives of at least two independent experiments. The plots of the red, green, and blue pixel intensities along the white arrow (in the middle panels) are presented. (**C**) Pearson’s correlation coefficient was determined by ImageJ (JACoP Plugin) for 15 cells in each treatment and presented as box and whiskers (min to max). Two-tailed t tests were performed (*, P ≤ 0.05; **, P ≤ 0.01; ***; P ≤ 0.001).
(PDF)

S3 Fig ORC2 and SIN3A but not MeCP2 co-localize with LANA dots in KSHV-infected cells. Immunofluorescence assays were performed to detect ORC2 (A) SIN3A (B) or MeCP2 (C) cellular localization in KSHV-negative (BJAB) and KSHV-positive (BCBL1) lymphoma cell lines.
The nucleus was stained with DAPI. Images are representatives of at least two independent experiments. Scale bar = 5 μm.
(PDF)

S4 Fig Expression of LANA and MeCP2 mutant proteins. NIH 3T3 cells were transfected with scFv-MeCP2, scFv-MeCP2 T158M, scFv-MeCP2 delMBD, and GFP-LANA expression vectors.
Cell extracts were subjected to SDS-PAGE and western blot analysis with anti-HA (upper panel), anti-LANA (middle panel), and actin (lower panel).
(PDF)

S5 Fig MBD deletion abrogates the ability of MeCP2 to recruit LANA. (A) SLK cells were transfected with dCas9-SunTag, scFv-MeCP2, GFP-LANA, and sgTelomere.
Different scFv-MeCP2 constructs were transfected to express wild-type (wt), T158M mutant, or a MBD deletion mutant of MeCP2, as illustrated on the left. An immunofluorescence assay detected scFv-MeCP2, or fluorescently labelled GFP-LANA. The nucleus was stained with DAPI. Scale bar = 5 μm. The plots of the red, green, and blue pixel intensities along the white arrow (in the middle panels) are presented. Images are representatives of at least three independent experiments. (**B**) Pearson’s correlation coefficient was determined for 15 cells in each treatment and presented as box and whiskers (min to max). Two-tailed *t* tests were performed (*, P ≤ 0.05; **, P ≤ 0.01; ***; P ≤ 0.001, ****; P ≤ 0.0001).
(PDF)

S1 Table Primers used in this study.
The table includes all primers used in this study.
(PDF)

S2 Table The values used to build graphs.
The table includes all the values that were used to generate graphs in this study.
(XLSX)

## References

[1] Henke-Gendo C, Schulz TF. Transmission and disease association of Kaposi’s sarcoma-associated herpesvirus: recent developments. Curr Opin Infect Dis. 2004;17(1):53–7. doi: 10.1097/00001432-200402000-00011 15090892

[2] Chang Y, Cesarman E, Pessin MS, Lee F, Culpepper J, Knowles DM, et al. Identification of herpesvirus-like DNA sequences in AIDS-associated Kaposi’s sarcoma. Science. 1994;266(5192):1865–9. doi: 10.1126/science.7997879 7997879

[3] Kedes DH, Lagunoff M, Renne R, Ganem D. Identification of the gene encoding the major latency-associated nuclear antigen of the Kaposi’s sarcoma-associated herpesvirus. J Clin Invest. 1997;100(10):2606–10. doi: 10.1172/JCI119804 9366576 PMC508462

[4] Rainbow L, Platt GM, Simpson GR, Sarid R, Gao SJ, Stoiber H, et al. The 222- to 234-kilodalton latent nuclear protein (LNA) of Kaposi’s sarcoma-associated herpesvirus (human herpesvirus 8) is encoded by orf73 and is a component of the latency-associated nuclear antigen. J Virol. 1997;71(8):5915–21. doi: 10.1128/JVI.71.8.5915-5921.1997 9223481 PMC191847

[5] Ye F, Lei X, Gao S-J. Mechanisms of Kaposi’s Sarcoma-Associated Herpesvirus Latency and Reactivation. Adv Virol. 2011;2011:193860. doi: 10.1155/2011/193860 21625290 PMC3103228

[6] Grundhoff A, Ganem D. The latency-associated nuclear antigen of Kaposi’s sarcoma-associated herpesvirus permits replication of terminal repeat-containing plasmids. J Virol. 2003;77(4):2779–83. doi: 10.1128/jvi.77.4.2779-2783.2003 12552022 PMC141125

[7] Hu J, Garber A, Renne R. The latency-associated nuclear antigen of Kaposi’s sarcoma-associated herpesvirus supports latent DNA replication in dividing cells. Journal of Virology. 2002;76(10):11677–87. 12388727 10.1128/JVI.76.22.11677-11687.2002 PMC136756

[8] Kelley-Clarke B, De Leon-Vazquez E, Slain K, Barbera AJ, Kaye KM. Role of Kaposi’s sarcoma-associated herpesvirus C-terminal LANA chromosome binding in episome persistence. J Virol. 2009;83(9):4326–37. doi: 10.1128/JVI.02395-08 19225000 PMC2668501

[9] De León Vázquez E, Kaye KM. The internal Kaposi’s sarcoma-associated herpesvirus LANA regions exert a critical role on episome persistence. J Virol. 2011;85(15):7622–33. doi: 10.1128/JVI.00304-11 21593163 PMC3147901

[10] Ballestas ME, Chatis PA, Kaye KM. Efficient persistence of extrachromosomal KSHV DNA mediated by latency-associated nuclear antigen. Science. 1999;284(5414):641–4. doi: 10.1126/science.284.5414.641 10213686

[11] Barbera AJ, Chodaparambil JV, Kelley-Clarke B, Joukov V, Walter JC, Luger K, et al. The Nucleosomal Surface as a Docking Station for Kaposi’s Sarcoma Herpesvirus LANA. Science. 2006;311(5762):856–61. doi: 10.1126/science.1120541 16469929

[12] Matsumura S, Persson LM, Wong L, Wilson AC. The Latency-Associated Nuclear Antigen Interacts with MeCP2 and Nucleosomes through Separate Domains. J Virol. 2010;84(5):2318–30. doi: 10.1128/jvi.01097-09 20032179 PMC2820923

[13] Krithivas A, Fujimuro M, Weidner M, Young DB, Hayward SD. Protein interactions targeting the latency-associated nuclear antigen of Kaposi’s sarcoma-associated herpesvirus to cell chromosomes. J Virol. 2002;76(22):11596–604. doi: 10.1128/jvi.76.22.11596-11604.2002 12388720 PMC136775

[14] Viejo-Borbolla A, Ottinger M, Brüning E, Bürger A, König R, Kati E, et al. Brd2/RING3 interacts with a chromatin-binding domain in the Kaposi’s Sarcoma-associated herpesvirus latency-associated nuclear antigen 1 (LANA-1) that is required for multiple functions of LANA-1. J Virol. 2005;79(21):13618–29. doi: 10.1128/JVI.79.21.13618-13629.2005 16227282 PMC1262589

[15] Hellert J, Weidner-Glunde M, Krausze J, Lünsdorf H, Ritter C, Schulz TF, et al. The 3D structure of Kaposi sarcoma herpesvirus LANA C-terminal domain bound to DNA. Proc Natl Acad Sci U S A. 2015;112(21):6694–9. doi: 10.1073/pnas.1421804112 25947153 PMC4450395

[16] Garber AC, Hu J, Renne R. Latency-associated nuclear antigen (LANA) cooperatively binds to two sites within the terminal repeat, and both sites contribute to the ability of LANA to suppress transcription and to facilitate DNA replication. J Biol Chem. 2002;277(30):27401–11. doi: 10.1074/jbc.M203489200 12015325

[17] Domsic JF, Chen H-S, Lu F, Marmorstein R, Lieberman PM. Molecular basis for oligomeric-DNA binding and episome maintenance by KSHV LANA. PLoS Pathog. 2013;9(10):e1003672. doi: 10.1371/journal.ppat.1003672 24146617 PMC3798644

[18] De Leo A, Deng Z, Vladimirova O, Chen H-S, Dheekollu J, Calderon A, et al. LANA oligomeric architecture is essential for KSHV nuclear body formation and viral genome maintenance during latency. PLoS Pathog. 2019;15(1):e1007489. doi: 10.1371/journal.ppat.1007489 30682185 PMC6364946

[19] Shamay M, Krithivas A, Zhang J, Hayward SD. Recruitment of the de novo DNA methyltransferase Dnmt3a by Kaposi’s sarcoma-associated herpesvirus LANA. Proc Natl Acad Sci U S A. 2006;103(39):14554–9. doi: 10.1073/pnas.0604469103 16983096 PMC1599998

[20] Hall JM, McDonnell DP, Korach KS. Allosteric regulation of estrogen receptor structure, function, and coactivator recruitment by different estrogen response elements. Mol Endocrinol. 2002;16(3):469–86. doi: 10.1210/mend.16.3.0814 11875105

[21] Nan X, Campoy FJ, Bird A. MeCP2 is a transcriptional repressor with abundant binding sites in genomic chromatin. Cell. 1997;88(4):471–81. doi: 10.1016/s0092-8674(00)81887-5 9038338

[22] Jones PL, Veenstra GJ, Wade PA, Vermaak D, Kass SU, Landsberger N, et al. Methylated DNA and MeCP2 recruit histone deacetylase to repress transcription. Nat Genet. 1998;19(2):187–91. doi: 10.1038/561 9620779

[23] Nan X, Ng HH, Johnson CA, Laherty CD, Turner BM, Eisenman RN, et al. Transcriptional repression by the methyl-CpG-binding protein MeCP2 involves a histone deacetylase complex. Nature. 1998;393(6683):386–9. doi: 10.1038/30764 9620804

[24] Bannister AJ, Zegerman P, Partridge JF, Miska EA, Thomas JO, Allshire RC, et al. Selective recognition of methylated lysine 9 on histone H3 by the HP1 chromo domain. Nature. 2001;410(6824):120–4. doi: 10.1038/35065138 11242054

[25] Lachner M, O’Carroll D, Rea S, Mechtler K, Jenuwein T. Methylation of histone H3 lysine 9 creates a binding site for HP1 proteins. Nature. 2001;410(6824):116–20. doi: 10.1038/35065132 11242053

[26] Agarwal N, Hardt T, Brero A, Nowak D, Rothbauer U, Becker A, et al. MeCP2 interacts with HP1 and modulates its heterochromatin association during myogenic differentiation. Nucleic Acids Res. 2007;35(16):5402–8. doi: 10.1093/nar/gkm599 17698499 PMC2018631

[27] Gonzales ML, Adams S, Dunaway KW, LaSalle JM. Phosphorylation of distinct sites in MeCP2 modifies cofactor associations and the dynamics of transcriptional regulation. Mol Cell Biol. 2012;32(14):2894–903. doi: 10.1128/MCB.06728-11 22615490 PMC3416191

[28] Jinek M, Chylinski K, Fonfara I, Hauer M, Doudna JA, Charpentier E. A programmable dual-RNA-guided DNA endonuclease in adaptive bacterial immunity. Science. 2012;337(6096):816–21. doi: 10.1126/science.1225829 22745249 PMC6286148

[29] Qi LS, Larson MH, Gilbert LA, Doudna JA, Weissman JS, Arkin AP, et al. Repurposing CRISPR as an RNA-guided platform for sequence-specific control of gene expression. Cell. 2013;152(5):1173–83. doi: 10.1016/j.cell.2013.02.022 23452860 PMC3664290

[30] Gilbert LA, Larson MH, Morsut L, Liu Z, Brar GA, Torres SE, et al. CRISPR-mediated modular RNA-guided regulation of transcription in eukaryotes. Cell. 2013;154(2):442–51. doi: 10.1016/j.cell.2013.06.044 23849981 PMC3770145

[31] Deng W, Shi X, Tjian R, Lionnet T, Singer RH. CASFISH: CRISPR/Cas9-mediated in situ labeling of genomic loci in fixed cells. Proc Natl Acad Sci U S A. 2015;112(38):11870–5. doi: 10.1073/pnas.1515692112 26324940 PMC4586837

[32] Chen B, Gilbert LA, Cimini BA, Schnitzbauer J, Zhang W, Li G-W, et al. Dynamic imaging of genomic loci in living human cells by an optimized CRISPR/Cas system. Cell. 2013;155(7):1479–91. doi: 10.1016/j.cell.2013.12.001 24360272 PMC3918502

[33] Anton T, Bultmann S, Leonhardt H, Markaki Y. Visualization of specific DNA sequences in living mouse embryonic stem cells with a programmable fluorescent CRISPR/Cas system. Nucleus. 2014;5(2):163–72. doi: 10.4161/nucl.28488 24637835 PMC4049922

[34] Duan J, Lu G, Hong Y, Hu Q, Mai X, Guo J, et al. Live imaging and tracking of genome regions in CRISPR/dCas9 knock-in mice. Genome Biol. 2018;19(1):192. doi: 10.1186/s13059-018-1530-1 30409154 PMC6225728

[35] Tanenbaum ME, Gilbert LA, Qi LS, Weissman JS, Vale RD. A protein-tagging system for signal amplification in gene expression and fluorescence imaging. Cell. 2014;159(3):635–46. doi: 10.1016/j.cell.2014.09.039 25307933 PMC4252608

[36] Kelley-Clarke B, Ballestas ME, Srinivasan V, Barbera AJ, Komatsu T, Harris T-A, et al. Determination of Kaposi’s sarcoma-associated herpesvirus C-terminal latency-associated nuclear antigen residues mediating chromosome association and DNA binding. J Virol. 2007;81(8):4348–56. doi: 10.1128/JVI.01289-06 17287261 PMC1866165

[37] Hellert J, Weidner-Glunde M, Krausze J, Richter U, Adler H, Fedorov R, et al. A structural basis for BRD2/4-mediated host chromatin interaction and oligomer assembly of Kaposi sarcoma-associated herpesvirus and murine gammaherpesvirus LANA proteins. PLoS Pathog. 2013;9(10):e1003640. doi: 10.1371/journal.ppat.1003640 24146614 PMC3798688

[38] Stedman W, Deng Z, Lu F, Lieberman PM. ORC, MCM, and Histone Hyperacetylation at the Kaposi’s Sarcoma-Associated Herpesvirus Latent Replication Origin. J Virol. 2004;78(22):12566–75. doi: 10.1128/jvi.78.22.12566-12575.2004 15507644 PMC525046

[39] Verma SC, Lan K, Choudhuri T, Cotter MA, Robertson ES. An autonomous replicating element within the KSHV genome. Cell Host Microbe. 2007;2(2):106–18. doi: 10.1016/j.chom.2007.07.002 18005725 PMC4287363

[40] Krithivas A, Young DB, Liao G, Greene D, Hayward SD. Human herpesvirus 8 LANA interacts with proteins of the mSin3 corepressor complex and negatively regulates Epstein-Barr virus gene expression in dually infected PEL cells. J Virol. 2000;74(20):9637–45. doi: 10.1128/jvi.74.20.9637-9645.2000 11000236 PMC112396

[41] Cai Q, Cai S, Zhu C, Verma SC, Choi J-Y, Robertson ES. A unique SUMO-2-interacting motif within LANA is essential for KSHV latency. PLoS Pathog. 2013;9(11):e1003750. doi: 10.1371/journal.ppat.1003750 24278015 PMC3836728

[42] Verma SC, Cai Q, Kreider E, Lu J, Robertson ES. Comprehensive analysis of LANA interacting proteins essential for viral genome tethering and persistence. PLoS One. 2013;8(9):e74662. doi: 10.1371/journal.pone.0074662 24040311 PMC3770571

[43] Stuber G, Mattsson K, Flaberg E, Kati E, Markasz L, Sheldon JA, et al. HHV-8 encoded LANA-1 alters the higher organization of the cell nucleus. Mol Cancer. 2007;6:28. doi: 10.1186/1476-4598-6-28 17433107 PMC1857702

[44] Rhee I, Bachman KE, Park BH, Jair K-W, Yen R-WC, Schuebel KE, et al. DNMT1 and DNMT3b cooperate to silence genes in human cancer cells. Nature. 2002;416(6880):552–6. doi: 10.1038/416552a 11932749

[45] Griffiths R, Whitehouse A. Herpesvirus saimiri episomal persistence is maintained via interaction between open reading frame 73 and the cellular chromosome-associated protein MeCP2. J Virol. 2007;81(8):4021–32. doi: 10.1128/JVI.02171-06 17267510 PMC1866103

[46] Dedon PC, Soults JA, David Allis C, Gorovsky MA. A simplified formaldehyde fixation and immunoprecipitation technique for studying protein-DNA interactions. Analytical Biochemistry. 1991;197(1):83–90. doi: 10.1016/0003-2697(91)90359-2 1952079

[47] Orlando V, Paro R. Mapping Polycomb-repressed domains in the bithorax complex using in vivo formaldehyde cross-linked chromatin. Cell. 1993;75(6):1187–98. doi: 10.1016/0092-8674(93)90328-n 7903220

[48] Gonzalez-Serricchio AS, Sternberg PW. Visualization of C. elegans transgenic arrays by GFP. BMC Genet. 2006;7:36. doi: 10.1186/1471-2156-7-36 16759392 PMC1539001

[49] Mughal N, Coppotelli G, Callegari S, Gastaldello S, Masucci MG. Interaction of gamma-herpesvirus genome maintenance proteins with cellular chromatin. PLoS One. 2013;8(5):e62783. doi: 10.1371/journal.pone.0062783 23667520 PMC3646995

[50] Gao Y, Han M, Shang S, Wang H, Qi LS. Interrogation of the dynamic properties of higher-order heterochromatin using CRISPR-dCas9. Mol Cell. 2021;81(20):4287-4299.e5. doi: 10.1016/j.molcel.2021.07.034 34428454 PMC8541924

[51] Shin Y, Chang Y-C, Lee DSW, Berry J, Sanders DW, Ronceray P, et al. Liquid Nuclear Condensates Mechanically Sense and Restructure the Genome. Cell. 2018;175(6):1481-1491.e13. doi: 10.1016/j.cell.2018.10.057 30500535 PMC6724728

[52] Vladimirova O, De Leo A, Deng Z, Wiedmer A, Hayden J, Lieberman PM. Phase separation and DAXX redistribution contribute to LANA nuclear body and KSHV genome dynamics during latency and reactivation. PLoS Pathog. 2021;17(1):e1009231. doi: 10.1371/journal.ppat.1009231 33471863 PMC7943007

[53] Wang L, Hu M, Zuo M-Q, Zhao J, Wu D, Huang L, et al. Rett syndrome-causing mutations compromise MeCP2-mediated liquid-liquid phase separation of chromatin. Cell Res. 2020;30(5):393–407. doi: 10.1038/s41422-020-0288-7 32111972 PMC7196128

[54] Adams VH, McBryant SJ, Wade PA, Woodcock CL, Hansen JC. Intrinsic disorder and autonomous domain function in the multifunctional nuclear protein, MeCP2. J Biol Chem. 2007;282(20):15057–64. doi: 10.1074/jbc.M700855200 17371874

[55] Ghosh RP, Horowitz-Scherer RA, Nikitina T, Shlyakhtenko LS, Woodcock CL. MeCP2 binds cooperatively to its substrate and competes with histone H1 for chromatin binding sites. Mol Cell Biol. 2010;30(19):4656–70. doi: 10.1128/MCB.00379-10 20679481 PMC2950531

[56] Traynor J, Agarwal P, Lazzeroni L, Francke U. Gene expression patterns vary in clonal cell cultures from Rett syndrome females with eight different MECP2 mutations. BMC Med Genet. 2002;3:12. doi: 10.1186/1471-2350-3-12 12418965 PMC137585

[57] Fujimuro M, Hayward SD. The latency-associated nuclear antigen of Kaposi’s sarcoma-associated herpesvirus manipulates the activity of glycogen synthase kinase-3beta. J Virol. 2003;77(14):8019–30. doi: 10.1128/jvi.77.14.8019-8030.2003 12829841 PMC161926

[58] Baker SA, Chen L, Wilkins AD, Yu P, Lichtarge O, Zoghbi HY. An AT-hook domain in MeCP2 determines the clinical course of Rett syndrome and related disorders. Cell. 2013;152(5):984–96. doi: 10.1016/j.cell.2013.01.038 23452848 PMC3641682

[59] Morita S, Noguchi H, Horii T, Nakabayashi K, Kimura M, Okamura K, et al. Targeted DNA demethylation in vivo using dCas9-peptide repeat and scFv-TET1 catalytic domain fusions. Nat Biotechnol. 2016;34(10):1060–5. doi: 10.1038/nbt.3658 27571369

[60] Stringer BW, Day BW, D’Souza RCJ, Jamieson PR, Ensbey KS, Bruce ZC, et al. A reference collection of patient-derived cell line and xenograft models of proneural, classical and mesenchymal glioblastoma. Sci Rep. 2019;9(1):4902. doi: 10.1038/s41598-019-41277-z 30894629 PMC6427001

[61] Shamay M, Greenway M, Liao G, Ambinder RF, Hayward SD. De novo DNA methyltransferase DNMT3b interacts with NEDD8-modified proteins. J Biol Chem. 2010;285(47):36377–86. doi: 10.1074/jbc.M110.155721 20847044 PMC2978566

[62] Schneider CA, Rasband WS, Eliceiri KW. NIH Image to ImageJ: 25 years of image analysis. Nat Methods. 2012;9(7):671–5. doi: 10.1038/nmeth.2089 22930834 PMC5554542

