# Supplementary material for: Unidirectional recruitment between MeCP2 and KSHV-encoded LANA revealed by CRISPR/Cas9 recruitment assay
Source: PLoS Pathog. 2025 Mar 10;21(3):e1012972. doi: 10.1371/journal.ppat.1012972 (PMC11913271; doi:10.1371/journal.ppat.1012972)
Supplement: S1 Fig — HEK 293T cells were transfected with scFv-MeCP2, scFv-MeCP2 T158M, scFv-MeCP2 delMBD, scFv-HP1, and scFv-HDAC1 (A) or scFv-LANA (B) expression vectors. Cell extracts were subjected to SDS-PAGE and western blot analysis. The scFv-fused proteins (upper panel) and beta-actin (lower panel) were detected with anti-HA and anti-beta actin antibodies, respectively. (PDF) [file ppat.1012972.s001.pdf]

Ido Lavi, et al.

**Unidirectional recruitment between MeCP2 and KSHV-encoded LANA revealed by CRISPR/Cas9 recruitment assay**

**Supplementary Data:**

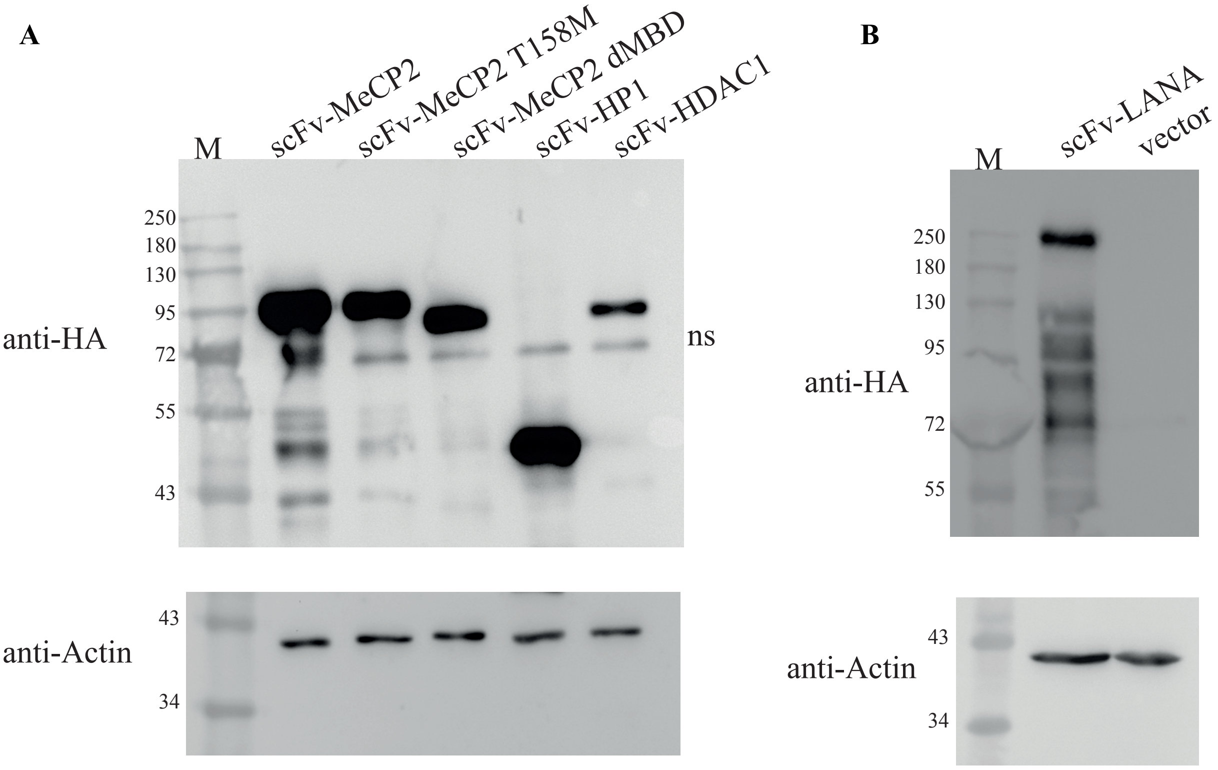


**S1 Fig. Expression of the fused proteins.** HEK 293T cells were transfected with scFv-MeCP2, scFv-MeCP2 T158M, scFv-MeCP2 delMBD, scFv-HP1, and scFv-HDAC1 (**A**) or scFv-LANA (**B**) expression vectors. Cell extracts were subjected to SDS-PAGE and western blot analysis. The scFv -fused proteins (upper panel) and beta-actin (lower panel) were detected with anti-HA and anti-beta actin antibodies, respectively.
