# Supplementary material for: Unidirectional recruitment between MeCP2 and KSHV-encoded LANA revealed by CRISPR/Cas9 recruitment assay
Source: PLoS Pathog. 2025 Mar 10;21(3):e1012972. doi: 10.1371/journal.ppat.1012972 (PMC11913271; doi:10.1371/journal.ppat.1012972)
Supplement: S2 Fig — HCT116 (A) and DKO (DNMT3B-/- and DNMT1 -/-) HCT116 cells (B) were transfected with dCas9-SunTag, scFv-LANA, and sgTelomere as illustrated on the left of the images. An immunofluorescence assay was performed to detect MeCP2 (green) and LANA (red). The nucleus was stained with DAPI. Scale bar = 5 μm. Images are representatives of at least two independent experiments. The plots of the red, green, and blue pixel intensities along the white arrow (in the middle panels) are presented. (C) Pearson’s correlation coefficient was determined by ImageJ (JACoP Plugin) for 15 cells in each treatment and presented as box and whiskers (min to max). Two-tailed t tests were performed (*, P ≤ 0.05; **, P ≤ 0.01; ***; P ≤ 0.001). (PDF) [file ppat.1012972.s002.pdf]

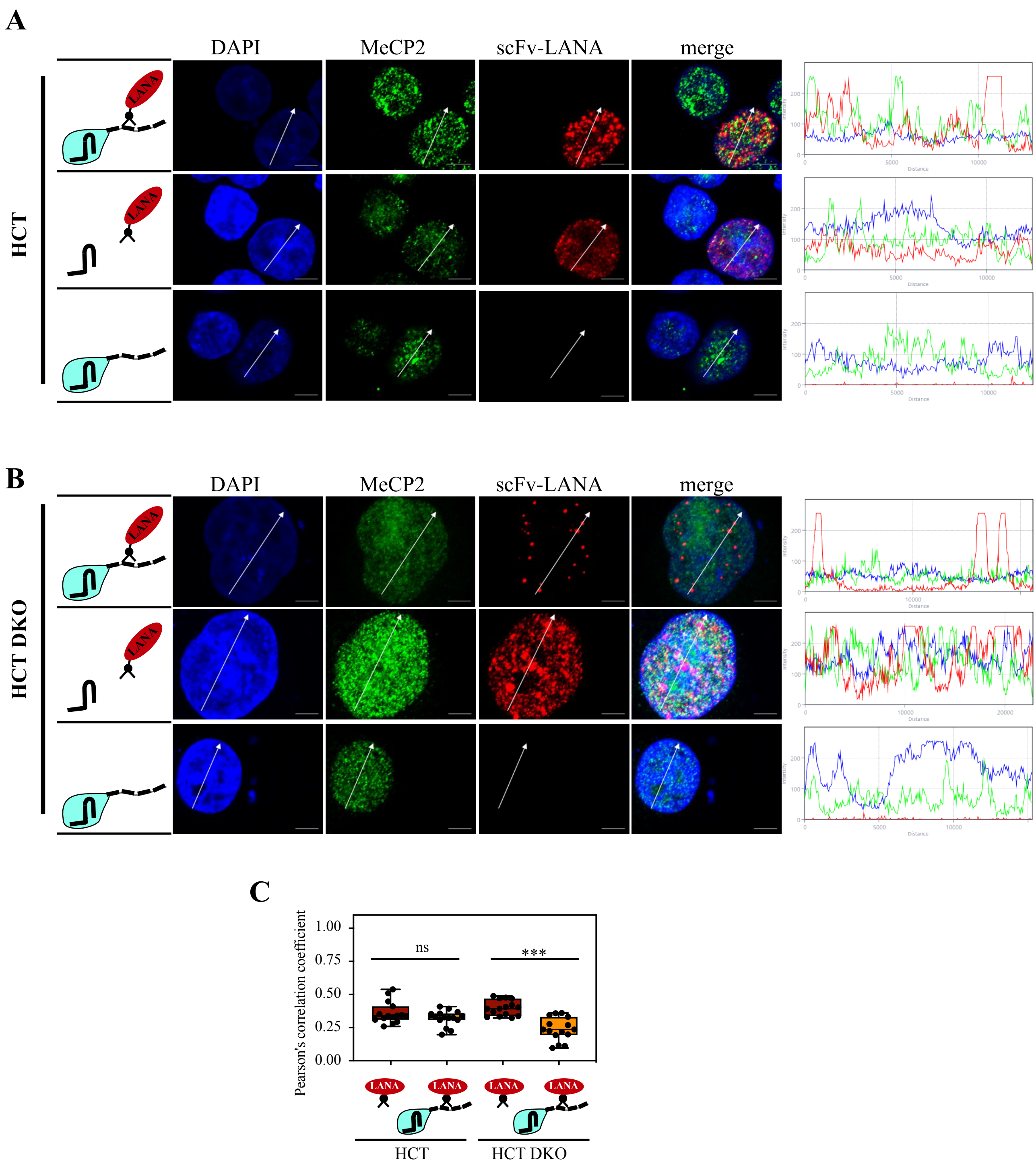

S2 Fig. LANA cannot recruit MeCP2 even in HCT-DKO cells. HCT116 (**A**) and DKO (DNMT3B-/- and DNMT1 -/-) HCT116 cells (**B**) were transfected with dCas9-SunTag, scFv-LANA, and sgTelomere as illustrated on the left of the images. An immunofluorescence assay was performed to detect MeCP2 (green) and LANA (red). The nucleus was stained with DAPI. Scale bar = 5 µm. Images are representatives of at least two independent experiments. The plots of the red, green, and blue pixel intensities along the white arrow (in the middle panels) are presented. (**C**) Pearson's correlation coefficient was determined by ImageJ (JACoP Plugin) for 15 cells in each treatment and presented as box and whiskers (min to max). Two-tailed t tests were performed (*, P ≤ 0.05; **, P ≤ 0.01; ***; P ≤ 0.001).
