# Supplementary material for: Unidirectional recruitment between MeCP2 and KSHV-encoded LANA revealed by CRISPR/Cas9 recruitment assay
Source: PLoS Pathog. 2025 Mar 10;21(3):e1012972. doi: 10.1371/journal.ppat.1012972 (PMC11913271; doi:10.1371/journal.ppat.1012972)
Supplement: S3 Fig — Immunofluorescence assays were performed to detect ORC2 (A) SIN3A (B) or MeCP2 (C) cellular localization in KSHV-negative (BJAB) and KSHV-positive (BCBL1) lymphoma cell lines. The nucleus was stained with DAPI. Images are representatives of at least two independent experiments. Scale bar = 5 μm. (PDF) [file ppat.1012972.s003.pdf]

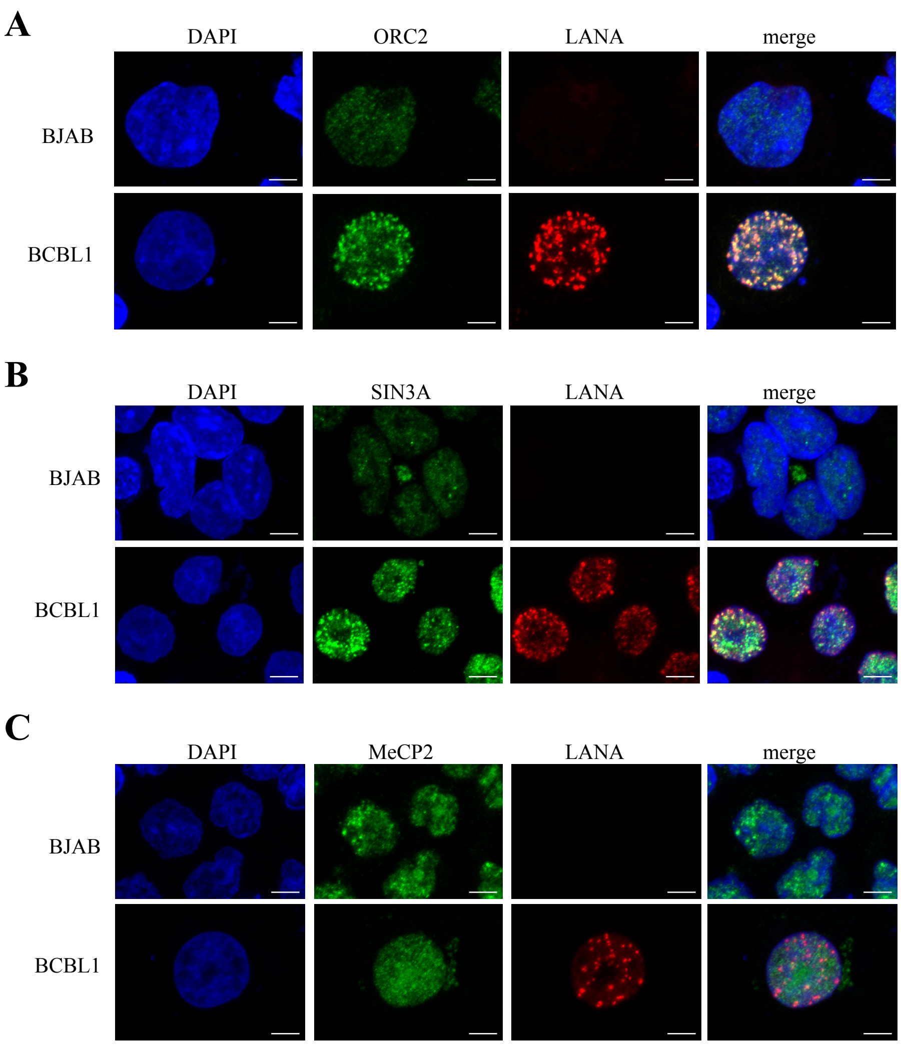

S3 Fig. ORC2 and SIN3A but not MeCP2 co-localize with LANA dots in KSHV infected cells. Immunofluorescence assays were performed to detect ORC2 (**A**) SIN3A (**B**) or MeCP2 (**C**) cellular localization in KSHV-negative (BJAB) and KSHV-positive (BCBL1) lymphoma cell lines. The nucleus was stained with DAPI. Images are representatives of at least two independent experiments. Scale bar = 5 µm.
