# Supplementary material for: Unidirectional recruitment between MeCP2 and KSHV-encoded LANA revealed by CRISPR/Cas9 recruitment assay
Source: PLoS Pathog. 2025 Mar 10;21(3):e1012972. doi: 10.1371/journal.ppat.1012972 (PMC11913271; doi:10.1371/journal.ppat.1012972)
Supplement: S4 Fig — NIH 3T3 cells were transfected with scFv-MeCP2, scFv-MeCP2 T158M, scFv-MeCP2 delMBD, and GFP-LANA expression vectors. Cell extracts were subjected to SDS-PAGE and western blot analysis with anti-HA (upper panel), anti-LANA (middle panel), and actin (lower panel). (PDF) [file ppat.1012972.s004.pdf]

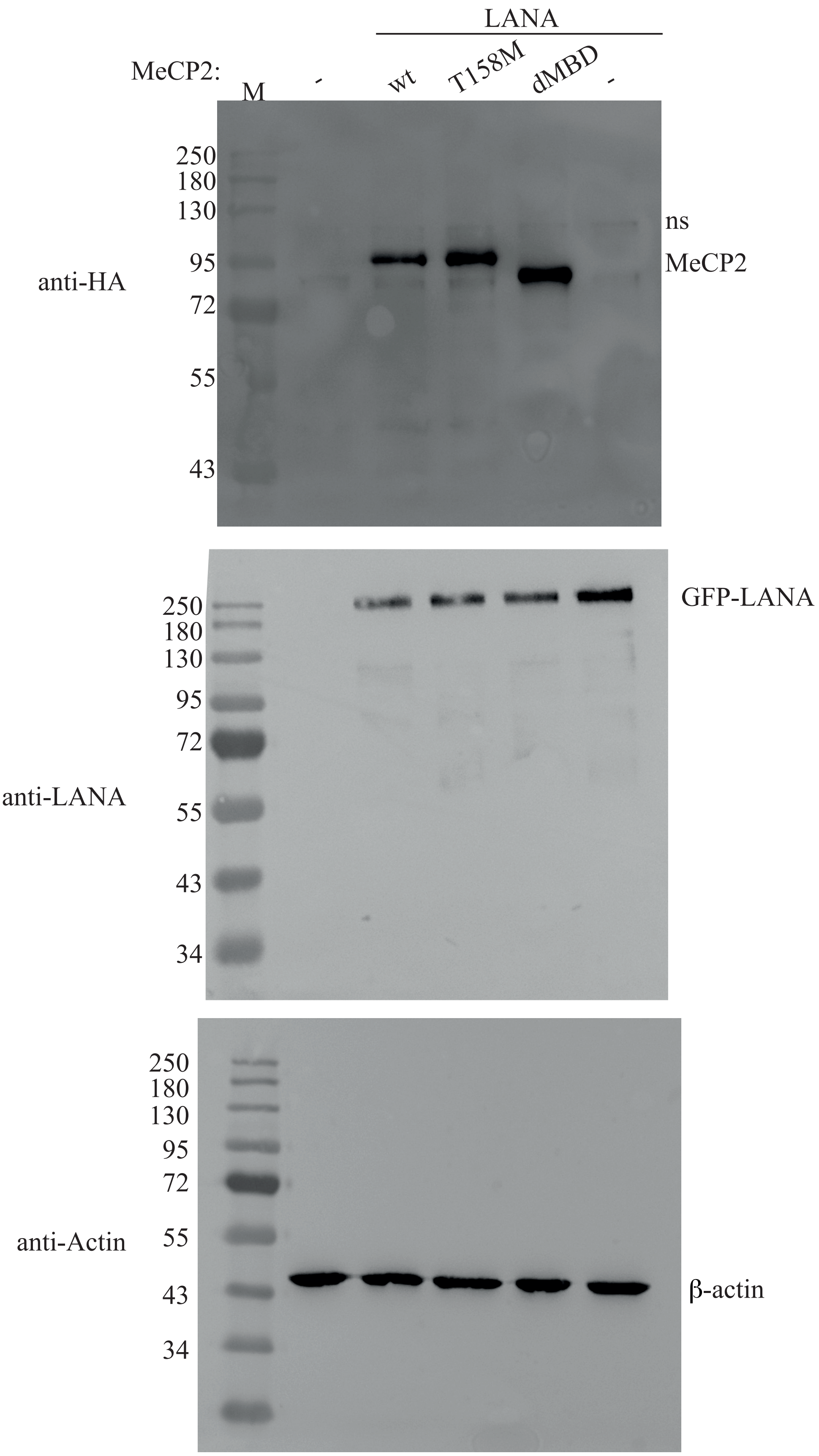

S4 Fig . Expression of LANA and MeCP2 mutant proteins. HEK 293T cells were transfected with scFv-MeCP2, scFv-MeCP2 T158M, scFv-MeCP2 delMBD, and GFP-LANA expression vectors. Cell extracts were subjected to SDS-PAGE and western blot analysis with anti-HA (upper panel), anti-LANA (middle panel) and actin (lower panel).
