# Supplementary material for: Unidirectional recruitment between MeCP2 and KSHV-encoded LANA revealed by CRISPR/Cas9 recruitment assay
Source: PLoS Pathog. 2025 Mar 10;21(3):e1012972. doi: 10.1371/journal.ppat.1012972 (PMC11913271; doi:10.1371/journal.ppat.1012972)
Supplement: S5 Fig — (A) SLK cells were transfected with dCas9-SunTag, scFv-MeCP2, GFP-LANA, and sgTelomere. Different scFv-MeCP2 constructs were transfected to express wild-type (wt), T158M mutant, or a MBD deletion mutant of MeCP2, as illustrated on the left. An immunofluorescence assay detected scFv-MeCP2, or fluorescently labelled GFP-LANA. The nucleus was stained with DAPI. Scale bar = 5 μm. The plots of the red, green, and blue pixel intensities along the white arrow (in the middle panels) are presented. Images are representatives of at least three independent experiments. (B) Pearson’s correlation coefficient was determined for 15 cells in each treatment and presented as box and whiskers (min to max). Two-tailed t tests were performed (*, P ≤ 0.05; **, P ≤ 0.01; ***; P ≤ 0.001, ****; P ≤ 0.0001). (PDF) [file ppat.1012972.s005.pdf]

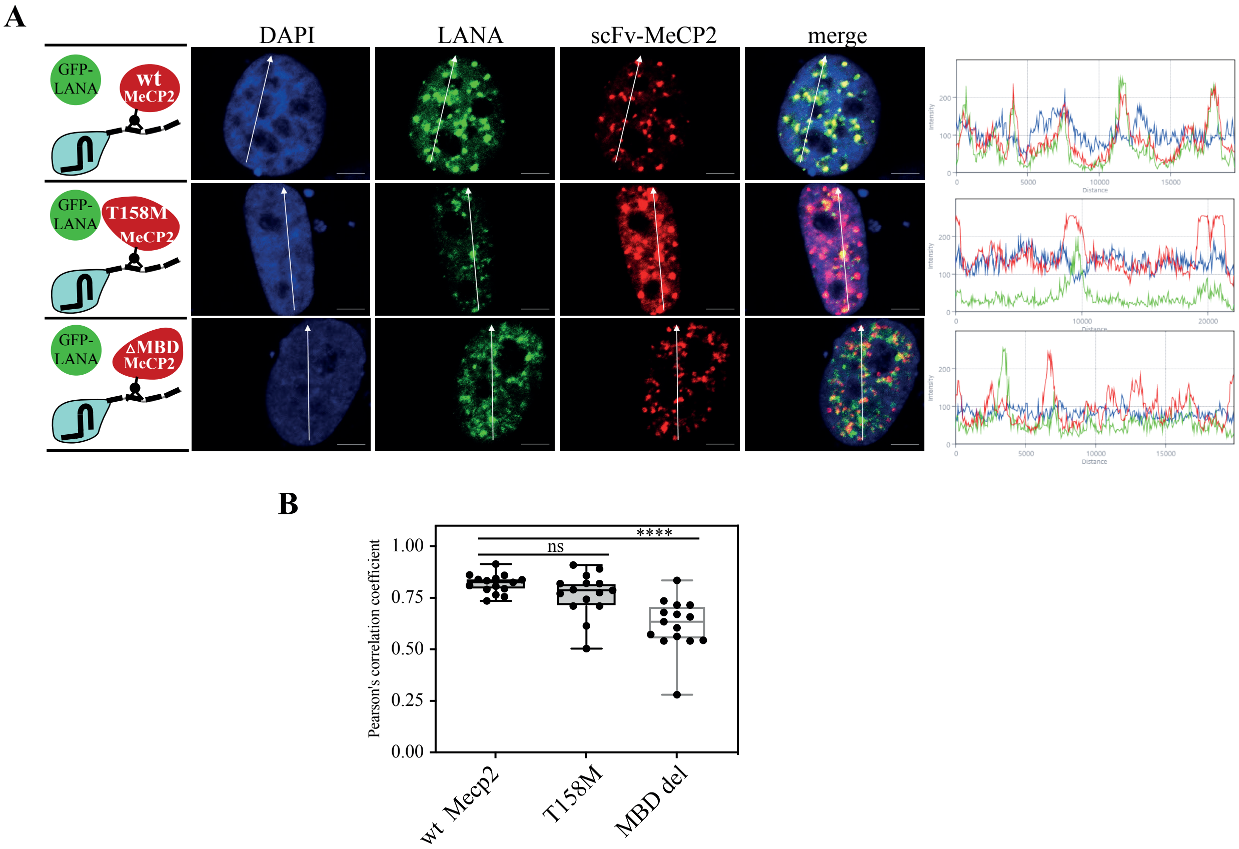

S5 Fig . MBD deletion abrogates the ability of MeCP2 to recruit LANA. (A) SLK cells were transfected with dCas9-SunTag, scFv-MeCP2, GFP-LANA, and sgTelomere. Different scFv-MeCP2 constructs were transfected to express wild-type (wt), T158M mutant, or a MBD deletion mutant of MeCP2, as illustrated on the left. An immunofluorescence assay detected scFv-MeCP2, or fluorescently labelled GFP-LANA. The nucleus was stained with DAPI. Scale bar = 5 μm. The plots of the red, green, and blue pixel intensities along the white arrow (in the middle panels) are presented. Images are representatives of at least three independent experiments. (**B**) Pearson's correlation coefficient was determined for 15 cells in each treatment and presented as box and whiskers (min to max). Two-tailed *t* tests were performed (*, $P \leq 0.05$; **, $P \leq 0.01$; ***; $P \leq 0.001$, ****; $P \leq 0.0001$).
