# Supplementary material for: Unidirectional recruitment between MeCP2 and KSHV-encoded LANA revealed by CRISPR/Cas9 recruitment assay
Source: PLoS Pathog. 2025 Mar 10;21(3):e1012972. doi: 10.1371/journal.ppat.1012972 (PMC11913271; doi:10.1371/journal.ppat.1012972)
Supplement: S1 Table — The table includes all primers used in this study. (PDF) [file ppat.1012972.s006.pdf]

S1 Table. Primers used in this study.

**Primer used for ChIP**	
**ERBB2a Chip F**	AGTCACCAGCCTCTGCATTTA
ERBB2A Chip R	CCAGCTTCACTTTCTCCCTCT
**Primer used for cloning**	
LANA mut F	gacgcgcTTTGGGAAAGGATGGAAGAC
LANA mut R	tataagcGATGTGTTGTGGCCTAGC
HDAC1 F	GATCCgATGGCGCAGACGCAGG
HDAC1 R	GCTCAGGCCAACTTGACCTCCT
HP1aF	GAGGGATCCGATGGGAAAGAAAACCAAGCGGAC
HP!a R	TCTGCGGCCGCTTAGCTCTTTGCTGT
MeCP2F	GAGGGATCCGATGGTAGCTGGGATGTT
MeCP2R	AGAGCGGCCGCCTAGCTAACTCTCTCG
MECP2 (T158M) F	gacacggaagcttaagcaaaggaaa
MECP2 (T158M) R	ttgggaatggcctgaggg
MBDdelFRAG1F	gcttgGATCCgatggtagctg
MBDdelFRAG1R	aggggctcccagaagcttccggcacagccg
MBDdelFRAG2F	gaagcttctgggagcccctcccgg
MBDdelFRAG2R	agtcgcGGCCGCct
**sgRNA sequences**	
sgRNA telomere	TTGGGTTAGGGTTAGGGTTAGGGTTAGTTTTAGAGCTAGAAATA GCAAGTTAAAATAAGGCTAGTCCGTTATCAACTTGAAAAAGTGG CACCGAGTCGGTGCTTTTTTC
sgRNA ERBB2 F	CACCGGTTGCCACTCCCAGACTTGT
sgRNA ERBB2R	AAACACAAGTCTGGGAGTGGCAACC
